# Quantifying species traits related to oviposition behavior and offspring survival in two important disease vectors

**DOI:** 10.1371/journal.pone.0239636

**Published:** 2020-09-25

**Authors:** Donald A. Yee, William C. Glasgow, Nnaemeka F. Ezeakacha

**Affiliations:** School of Biological, Environmental, and Earth Sciences, University of Southern Mississippi, Hattiesburg, MS, United States of America; Al-Azhar University, EGYPT

## Abstract

Animals with complex life cycles have traits related to oviposition and juvenile survival that can respond to environmental factors in similar or dissimilar ways. We examined the preference-performance hypothesis (PPH), which states that females lacking parental care select juvenile habitats that maximize fitness, for two ubiquitous mosquito species, *Aedes albopictus* and *Culex quinquefasciatus*. Specifically, we examined if environmental factors known to affect larval abundance patterns in the field played a role in the PPH for these species. We first identified important environmental factors from a field survey that predicted larvae across different spatial scales. We then performed two experiments, the first testing the independent responses of oviposition and larval survival to these environmental factors, followed by a combined experiment where initial oviposition decisions were allowed to affect larval life history measures. We used path analysis for this last experiment to determine important links among factors in explaining egg numbers, larval mass, development time, and survival. For separate trials, *Aedes albopictus* displayed congruence between oviposition and larval survival, however *C*. *quinquefasciatus* did not. For the combined experiment path analysis suggested neither species completely fit predictions of the PPH, with density dependent effects of initial egg number on juvenile performance in *A*. *albopictus*. For these species the consequences of female oviposition choices on larval performance do not appear to fit expectations of the PPH.

## Introduction

A seminal goal of ecology is to identify rules that determine the composition of a community in a specific place, at a specific time. Although classic ideas about the mechanisms that explain such patterns still remain valid (e.g., assembly rules and biotic interactions; [1, 2]), new ideas and syntheses are also being developed (reviewed in [3]). One area of interest is trait-mediated effects, especially as they relate to individual species distribution and abundance. Traits here are defined as attributes of individuals or species whose variation can have consequences for fitness [4], and in animals with complex life cycles may include traits related to egg-laying and juvenile survival. Such traits are often considered to be used to optimize fitness under the preference–performance hypothesis (or optimal oviposition theory). This hypothesis states that organisms lacking parental care would be expected to place their eggs in the most suitable location for their offspring, and selection should favor oviposition behavior that optimizes offspring performance [5, 6]. For many aquatic organisms, like insects and amphibians, oviposition behavior is determined by a variety of cues from the environment that have obvious consequences for adult fitness [7]. However, choices made by females are not the sole mechanism that explain species distribution patterns, as survival of juveniles may also vary across sites and can even be at odds with maternal choices. In insects, oviposition preferences and larval performance are well studied [5, 8], and can lead to congruence between larval survival and oviposition patterns on host plants [9]. However, female oviposition choice and larval survival do not always match (e.g., [10]).

Oviposition is an important factor explaining species presence and population sizes for many animals, including insects and amphibians, whose juveniles often occupy aquatic systems but where adults may be found across different habitat types [11]. Among organisms that use lentic systems for population production mosquitoes are often ubiquitous, abundant, and diverse [12]. Mosquitoes possess complex sensilla for detecting water-borne cues, can be found across a wide variety of habitat types, and have been used as a model organisms to explore questions about female egg laying patterns related to the presence of predators (e.g., [13, 14]), conspecifics (e.g., [15]), and various abiotic factors (e.g., [16]). Oviposition patterns are often context dependence, i.e., where biotic and abiotic conditions alter the nature or strength of population interactions [15]. Specifically oviposition patterns can be affected by the presence of predators in container systems [17], and can represent a major non-lethal effect of predators on prey [15].On a practical level, forecasting the spatial and temporal patterns of populations of mosquitoes is a great challenge and a valuable scientific endeavor for the implementation of effective vector control and public health strategies [18]. Environmental factors can act on any number of species traits, although for many aquatic insects like mosquitoes oviposition and larval survival are likely paramount. Various species-specific cues related to oviposition would constitute the traits for egg laying, whereas factors like food quantity and quality, tolerance to abiotic parameters, and interactions with other individuals would constitute those related to survival traits for larvae.

Female mosquitoes use a variety of sensory cues to locate potential oviposition sites, including olfactory (e.g., volatiles from the aquatic habitat), tactile (e.g., container surfaces), and visual (e.g., color) cues [12, 19]. These cues can be associated with environmental factors, including the density of bacteria [20], inorganic salts [12], detritus [21], or predators [22, 23]. Other factors may affect the presence of females near an oviposition site (e.g., terrestrial vegetation [24]). Besides oviposition, larval survival (and thus the adults produced from aquatic habitats) may be affected by a number of environmental factors. After hatching, survival of larvae depends on the availability of food resources (detritus, microorganisms [25]), severity of intra- and interspecific interactions (competition, predation [15, 26]), and tolerances to physical factors (e.g., temperature, salinity [12]). Thus, production of adults from a small aquatic container like a tire or tree hole is likely a consequence of selection of that container by gravid females and the quality of that location for larvae. For instance, salinities preferred for oviposition by *Culex quinquefasciatus* and *C*. *sitiens* do not necessarily correspond to environments capable of producing larvae [27], suggesting that the same environmental variables do not have a consistent effect across the mosquito life cycle. Such disconnects between oviposition and larval survival have also been shown to occur for the container-breeding species *Aedes albopictus* and *A*. *triseriatus* [28]. The idea that some factors may act to affect different aspects of the life cycle of an animal is an underexplored area in ecology, and as the majority of species on Earth have complex life cycles (e.g., most insects, amphibians, many marine invertebrates [29]) an thus an examination of the relationship between oviposition and larvae survival should prove fruitful to our understanding of community patterns in these animals in nature.

For mosquitoes in the genera *Culex* and *Aedes* there are inherent differences in traits related to oviposition and larval behavior. *Culex* spp. lay eggs on the water’s surface in free-floating rafts, which hatch soon after oviposition; *Aedes* spp. often lay eggs above the water line and these eggs hatch only when inundated [12]. One consequence of this difference is that gravid female *Culex* may respond to the immediate conditions of a habitat (e.g., food, larval density), whereas *Aedes* use a bet-hedging strategy to maximize future larval success [14]. Also, female *Aedes* are capable of laying eggs in multiple locations from a single gonotrophic cycle (i.e., skip oviposition), whereas *Culex* typically lay all their eggs in a single raft in one container [12]. Larvae of both genera feed on microorganisms [25], although *Culex* tend to filter feed on microorganisms from the water column with *Aedes* concentrating on browsing surfaces [25, 30]. Some *Culex* are associated with environments with high organic material or are found in highly contaminated bodies of water, a fact that seems to fit with their ability to tolerate extreme values of pH, salinity, and temperature [21, 31]. In contrast, *Aedes*, such as *A*. *albopictus*, [32] show reduced survival when exposed to pollution derived from other larvae. These genera also seem to differ in their associations with human habitation, with many *Culex* spp. being considered non-peridomestic [31, 33], and many *Aedes* spp. being considered peridomestic [34, 35]. The differences in traits related to oviposition and larval survival make these intriguing organisms to test hypotheses about population patterns in nature.

In this study we examined two medically important mosquito species: the Asian tiger mosquito *Aedes albopictus* (Skuse) and the southern house mosquito *Culex quinquefasciatus* (L); this latter species is part of a wider complex and can be found in the northern U.S. as *Culex pipiens* [31]. *Aedes albopictus* is a successful invasive species, now occurring across the eastern U.S. and on all continents except Antarctica and mainland Australia [36, 37]). *Culex quinquefasciatus* is a common species found in both small containers [38] and open water habitats [12]. Beyond these specific attributes, we chose these species for three reasons. First, like other small insects with complex life cycles, they represent good test subjects to examine trait-mediated effects. Second, these species vary in terms of their habitat selectivity, with larval *Aedes albopictus* found in small aquatic containers, and larval *Culex quinquefasciatus* found in containers and open water habitats [12]. Finally, beyond their ecological role as decomposers [e.g., 39] and food sources for other animals [e.g., 40], they also are important for the transmission of several important arboviruses: *A*. *albopictus* for arbovirus like dengue and Zika [41] and *C*. *quinquefasciatus* for West Nile virus [42].

We conducted a series of controlled field and laboratory experiments to examine how oviposition and larval survival of these species would respond to specific environmental factors. We hypothesized that because there can be inconsistencies between the effects of environmental factors on oviposition and larvae survival (e.g., [27]), that success of a species would be a consequence of the combined responses at both the juvenile (larval survival) and adult (eggs laid) stages. We predicted that similar responses to the same factor across the life cycle should be the norm, as success of offspring would depend on females making egg laying choices that benefit her offspring. This would be consistent with the preference–performance hypothesis [5, 6], which has been tested for several mosquitoes (e.g., [16, 43]), with varying support. Our approach was first, using an extensive field data set collected from an earlier project [38], to identify important environmental factors that were correlated with larvae of these species in containers across different spatial scales (six sites across three counties across the State of Mississippi). Second, we tested for responses of oviposition and larval survival to these environmental factors, separately, for each species. Finally, we ran a combined experiment by allowing initial oviposition decisions to affect larval responses, and then quantified the effects on specific life history traits. This final approach used structural equation modeling (Path Analysis) to determine important links among factors in explaining the numbers of eggs and larval mass, development time, and survival.

## Materials and methods

### Mosquitoes

For all experiments, we used lab-reared larvae and adult *Aedes albopictus* and *Culex quinquefasciatus* generated from eggs and larvae collected from aquatic habitats in and around Hattiesburg, MS. Adults were maintained in a colony room at 27 ºC on a 14:10 h L:D cycle with 1 h of dawn and 1 h of twilight and were provided with a cotton pad soaked with 10% sugar solution. Eggs were produced from adults that were blood-fed using Japanese quail, *Coturnix japonica* (Institutional Care and Use Committee #11092207 at the University of Southern Mississippi approved this work). Larvae of each species were separately eared in pans filled with reverse osmosis (RO) water and fed a diet of a 1:1 ratio by weight of lactalbumin-yeast powder (0.15 g on days 1, 4, and every other day thereafter) until pupation. Pupae were isolated and transferred into species-specific colony cages to develop into adults. *Aedes albopictus* were provided black cups lined with brown paper and filled to 2.5 cm with reverse osmosis (RO) water for oviposition, and *C*. *quinquefasciatus* were provided black bowls filled to 2.5 cm with larval rearing water. Eggs were used to establish new colonies. Larvae used in experiments were F_1_-F_4_, although we used a mixture of F_1_ and F_7_ *Aedes albopictus* eggs in pine survival experiments to achieve the numbers per replicate.

### Environmental factors

Specific environmental factors were selected to test oviposition responses and larval survival rates based on data collected on mosquito larvae in tires from across the state of Mississippi [38]. Briefly, data were collected from existing vehicle tires at six sites distributed across the state in a north-south fashion. Each site was sampled three times during 2012, thus generating 18 observations (6 sites x 3 sampling periods). As that study measured 14 environmental factors (i.e., tire diameter, canopy openness, water depth, water volume, water temperature, pH, protozoan richness, protozoan abundance, animal detritus, leaf detritus, plant reproductive detritus, pine detritus, wood detritus, find detritus, details of these methods can be found in [38]) that were important for mosquito populations, we needed an approach to narrow this list down to fewer factors to examine here. Thus we performed a series of step-wise multiple regression analyses at three spatial/temporal scales: each site for each time period (18 regressions), each time period regardless of site (3 regressions), and the overall data set regardless of time or site (1 regression). For each multiple regression, the significant factor(s) that explained larval populations were identified based on step-wise selection, and then to assess which factors were important to predicting each species, we examined the number of significant regressions at the site level (18), time period (3), and overall (1) (S1 Table). Thus, a factor was deemed important if it was found as part of multiple significant stepwise regression models at multiple sites, across time periods, and in the overall data set. Our initial analysis used a traditional significance threshold of P < 0.05, however it only produced one clear factor per species for us to examine (pine for *Ae*. *albopictus*, volume for *Cx*. *quinquefasciatus*). We then applied a more liberal threshold (P < 0.10) and that produced two factors for each species: *Aedes albopictus* water volume (4 sites, 1 time period, 0 overall) and pine detritus (2, 2, 1), and for *Culex quinquefasciatus* water volume (4, 3, 1) and canopy openness (4, 2, 1) (S1 Table). Regardless of these analysis, these factors are important in explaining larval presence for these species based on past work, and specifically are consistent with effects of water volume [38] and detritus [38, 44] for *Aedes albopictus* and water volume [38, 44] and canopy openness [38] for *Culex quinquefasciatus*.

### Oviposition experiments

The oviposition responses to environmental factors for each species were conducted at the University of Southern Mississippi Science Park (31º 21’12” N, 89º 21’35” W). Experiments occurred outside in large wooden screen boxes (hereafter, arrays). Each array was 2.4 m long x 1.2 m high and 1.2 m wide, constructed with pressure treated lumber, and covered on the sides with no-see-um mesh (Phifer Inc., Tuscaloosa, AL); the top was covered with clear plastic sheeting, had a concrete bottom, with a door at one end. Each array had a small segment of PVC attached to one side affixed with a stocking net sleeve to introduce adult mosquitoes. Inside each array (n = 4) we placed 8 automobile tires (rim size 16”, mean size from [38]) vertically along the perimeter. Each tire was provisioned with one or more factors depending on the experiment and washed with a 10% bleach solution and rinsed before experiments. Each array received four sets of artificial potted plants placed in cups of sand between pairs of tires, to provide additional adult resting sites. Each array also held 20 ml glass vials of 10% sucrose solution administered with a cotton wick as a food source for adult mosquitoes. Gravid females were introduced after all treatments, artificial plants, and sugar had been placed inside and the door sealed.

#### Pine detritus

Senescent pine needles were collected from the Lake Thoreau Environmental Center, located 13 km from the USM campus (31°19’37.63” N, 89°17’25.22” W) and dried at 50°C for ≥ 48 hrs. Dry needles were weighed and placed into tires containing 2000 ml of water and 200 ml of strained inoculum water (collected and homogenized from several undisturbed tires and strained to removed invertebrates), and allowed to exist outside for 3 d prior to the start of experiments. The range of pine detritus reflected values collected from natural tires [38]: 0, 14, 26, 38, 50, 62, 74, and 86 g. Tires were lined with brown paper as an oviposition substrate. We released 20 gravid female *A*. *albopictus* into each array and after 48 hrs collected papers to count eggs. Four replicates were conducted, and detritus levels were haphazardly assigned to tires.

#### Volume

This factor was important for *A*. *albopictus* and *C*. *quinquefasciatus*, and thus the design was the same for both species. We used eight volumes that spanned the range found in [38]: 0.1, 1.2, 2.4, 3.6, 4.8, 6.0, 7.2, and 8.4 L. The lowest volume corresponded to the lowest volume where larvae were collected [38]. Water added to tires consisted of a mixture of tap water and tire inoculum (prepared by taking a mixture of various detritus types and soaking in a 20 L bucket outside for several days). For each volume, we added 10 ml of inoculum for every 100 ml of volume (e.g., 1.2 L would receive 120 ml tire inoculum). Because *Culex* lay eggs in rafts on the water surface, experiments involving that species did not include eggs papers. All other elements of design were the same as for pine detritus.

#### Canopy cover

*Culex quinquefasciatus* trials involving variation in canopy cover used 2000 ml of water with 200 ml inoculum in each tire. Variation in cover was achieved using segments of black no-see-um mesh placed on the array above each tire to decrease light penetration. We first measured light intensity next to two arrays at several times during each of a cloudless and overcast day using a hand-held light meter (Extech Light Meter Model 401025, Nashua, NH). We used the mean of these two readings as the highest light level, then reduced the light by using more layers of black no-see-um mesh, with each layer reducing the level by ~50–60%. By using different layers of mesh, this allowed us to produce eight different levels of LUX (2400, 1200, 600, 300, 240, 180, 120, 60; higher values equate to more light). As above, all other elements of design were the same as for volume.

#### Statistical analyses

As oviposition responses to treatment levels were not independent (i.e., egg placement could affect the placement of other eggs in a tire) we used a mixed model randomized block design for each species and treatment, separately using PROC MIXED in SAS [45]. For the egg oviposition experiments we included array as a random factor in the analysis. To meet assumptions of normality and homoscedasticity, the number of eggs of *A*. *albopictus* were log_(x+1)_ transformed for volume. For *C*. *quinquefasciatus*, we used a 1/(x+1) transformation of egg rafts for volume, and log_(x+1)_ for canopy cover. Differences in egg or raft numbers among treatment levels were identified using a Tukey-Kramer HSD post-hoc test for multiple comparisons. All analyses were performed using SAS [45].

### Larval survival experiments

To assess the effects of our field parameters on larval survival, we also conducted laboratory experiments for both species using the same factors as those for oviposition. In all cases, we used black rubber bowls made of recycled tires (Miller Manufacturing, Glencoe, MN) to rear larvae to mimic the environment of tires. Bowls were kept in environmental chambers set at 27° C on a 14:10 light/dark schedule. For each replicate within a trial 90 1^st^ instar *Aedes albopictus* were used whereas replicates for *C*. *quinquefasciatus* used 55 1^st^ instars; these numbers reflect the mean number of larvae per tires from field observations [38]. In all cases, we used pupation as a surrogate for survival. The experiment lasted until all larvae had pupated or died.

#### Volume

The treatments consisted of rubber bowls filled with different volumes of water as for oviposition experiments (0.1, 1.2, 2.4, 3.6, 4.8, 6.0, 7.2, and 8.4 L). Small bowls (2.5 L) were used for the two lowest volumes, whereas larger bowls (8.5 L) were used for the remaining volumes. Each bowl was filled with 95% by volume RO water and 5% by volume inoculum. After the water was placed in the bowls, 0.25g of freeze-dried animal (crickets, *Gryllodes sigillatus*, Fluker Farms, Port Allen, LA) detritus was placed in each bowl to provide food for developing larvae. Bowls were left in incubators for three days to encourage the growth of the microbial community before the addition of larvae.

#### Pine detritus

Senescent pine needles were obtained from Lake Thoreau and dried at 50°C for 48 hrs before establishing treatment levels identical to those used in the oviposition trials (0, 14, 26, 38, 50, 62, 74, and 86 g). Detritus was placed in individual 2.5 L bowls with 2.0 L RO water and 0.2 L tire inoculum strained with a 150-micron sieve. Detritus sat for 48 hrs before larvae were added. Larvae *A*. *albopictus* densities were identical as in the volume levels.

#### Canopy cover

Small bowls were filled with 2.0 L of water (1.95 L RO water, 0.05 L inoculate water). The highest light level within incubators registered at 1500 LUX and each subsequent layer of no-see-um mesh saw a decrease in light by ~50% (1500, 750, 375,180, 90, 45, 20, 10 LUX). Thus, this gradient of light levels was proportionally similar to but lower than those used in the oviposition trials. Detritus, soaking duration, larval *C*. *quinquefasciatus*, and other factors were identical as in the volume levels.

#### Statistical analyses

We assessed differences among treatment levels for mosquito survival using 1-way ANOVA. To meet assumptions of normality and homoscedasticity, survival of *A*. *albopictus* was square root +1 transformed for volume. For *C*. *quinquefasciatus*, we used a log_(x+1)_ transformation for survival for volume and canopy cover. Differences in mosquito survival and oviposition among treatment levels were identified using the Tukey-Kramer HSD post-hoc test for multiple comparisons where applicable.

### Linking oviposition to life history and survival

We performed a combined experiment to test the hypothesis that oviposition responses by females have consequences for life history and survival of progeny. Specifically, we repeated the oviposition experiment for each species but crossed each environmental factor within each array. For *A*. *albopictus*, we used three volumes (2.4, 4.8, 8.4 L) crossed with three levels of pine detritus (0, 14, 62 g). We excluded the 0 g pine detritus by 2.4 L volume, leaving us eight total combinations. For *C*. *quinquefasciatus*, we used three volumes (0.1, 2.4, 6.0 L) and canopy cover (10, 750, 1550 LUX). Here, we excluded the 0.1 L by 10 LUX combination. Values chosen for both species generally elicited the highest oviposition responses of females during the separate oviposition trials (see Results). Because we planned to quantify different life history traits, we used 40 females during these trials to generate as many eggs as possible. We used the same general methods as in the separate oviposition trials (e.g., artificial plants, oviposition water, sugar). After 48 hrs, we counted eggs and rafts as before, but unlike the oviposition trials, here we returned larvae to the arrays and followed their development. Specifically, we hatched eggs of both species and placed offspring in 8.5 L rubber bowls within each upright tire. This was done to facilitate the identification and removal of pupae but to maintain the same microenvironment as the tire. *Aedes* egg papers were kept out of water for one week to allow for embryonation, whereas *C*. *quinquefasciatus* eggs, which hatch within 24 hrs, were immediately placed into bowls after counting emerging larvae. In all cases, we used the number of hatched larvae added to bowls to reflect female reproductive output. For treatment combinations that lacked food for developing larvae (e.g., all *C*. *quinquefasciatus* treatments, no pine treatment levels for *A*. *albopictus*), we added 0.25 g of crickets to each bowl. We ran four replicates of each species with two replicates run for each species during each trial (two trials were run to achieve our four replicates per species). All bowls were monitored every other day for the presence of pupae, which when present were removed and isolated in 0.25-dram shell vials until they enclosed. Adults were then sexed, identified to species, and then dried at 50º C for > 48 hrs and weighed to the nearest 0.0001 mg using an XP2U ultra-microbalance (Mettler Toledo Inc., Columbus, Ohio). For each bowl, we determined the mean female weight (mg), female development time (from egg to adult), and percent survival (males were excluded from weight and development times given that they are not the main driver of population sizes in mosquitoes as they don’t lay eggs).

Oviposition patterns were linked to mosquito life history (female mass, development time) and survival using path analysis, which can test for the effects of multiple independent variables on multiple dependent variables [46]. In path analysis, unlike multiple regression, a variable may be both dependent (i.e., affected by other variables) and independent (i.e., affecting other variables). Path coefficients, like standardized regression coefficients, quantify direct effects on a dependent variable caused by variation in an independent variable, independently of direct effects of other independent variables [47, 48]. The importance of a path was evaluated by testing the fit of reduced models in which paths are removed, relative to a full model [47, 48]. Reduced models were compared to the full model using goodness-of-fit χ^2^ tests (PROC CALIS; [45]). We hypothesized three sets of paths based on the relationship between oviposition and larval survival in explaining life history parameters and survival: a Combined Model (Fig 4A), a Larval Model (Fig 4B), and an Oviposition Model (Fig 4C). The Combined model served as the full model, in which all variables were connected to all other variables. Moreover, if this model was supported in any way, it would suggest that both oviposition (i.e., number of hatched larvae) and larval survival and life history traits (female mass and development time) were both affected by habitat factors (i.e., ecological filters are consistent and important for both life stages). The Larval model contained direct effects of environmental factors on survival, female mass, and female development time. If this model was supported then it would suggest that habitat parameters largely worked to affect larval traits, but that egg laying was less important. The Oviposition model contained only the direct paths between environmental factors and the number of hatched larvae produced, and if supported, would suggest that filters only acted on females for each species. After analyzing the Combined model, paths were then removed in a systematic way, and if removing a link did not decrease the fit of the model, then it was assumed that the link was unimportant in explaining variation in the dependent variable. If this occurred, the path was eliminated and the next path was tested. Briefly, paths were removed between independent variables and eggs, then between independent variables and life history traits, and then between eggs and life history traits. We also ran the same model using λ’, a composite index that acts as a surrogate of population growth [49] in place of survival, female mass, and development time. Unlike other studies that have considered only population growth (e.g., [33, 50]) we chose to focus on the individual life history variables for two reasons. First, the results were redundant in that we found similar results for λ’ to using each life history variable and survival, separately (not shown). And second, as traits could be affected independently by the environmental factors, using a surrogate that incorporated all three might mask important patterns. The final model for each species thus will represent all important paths included in the model to explain variation in oviposition and larval survival and could then be compared to our three hypothesized models to assess the importance of ecological filters in shaping patterns of these important insects.

## Results

### Oviposition and larval survival experiments

**Fig 1:**
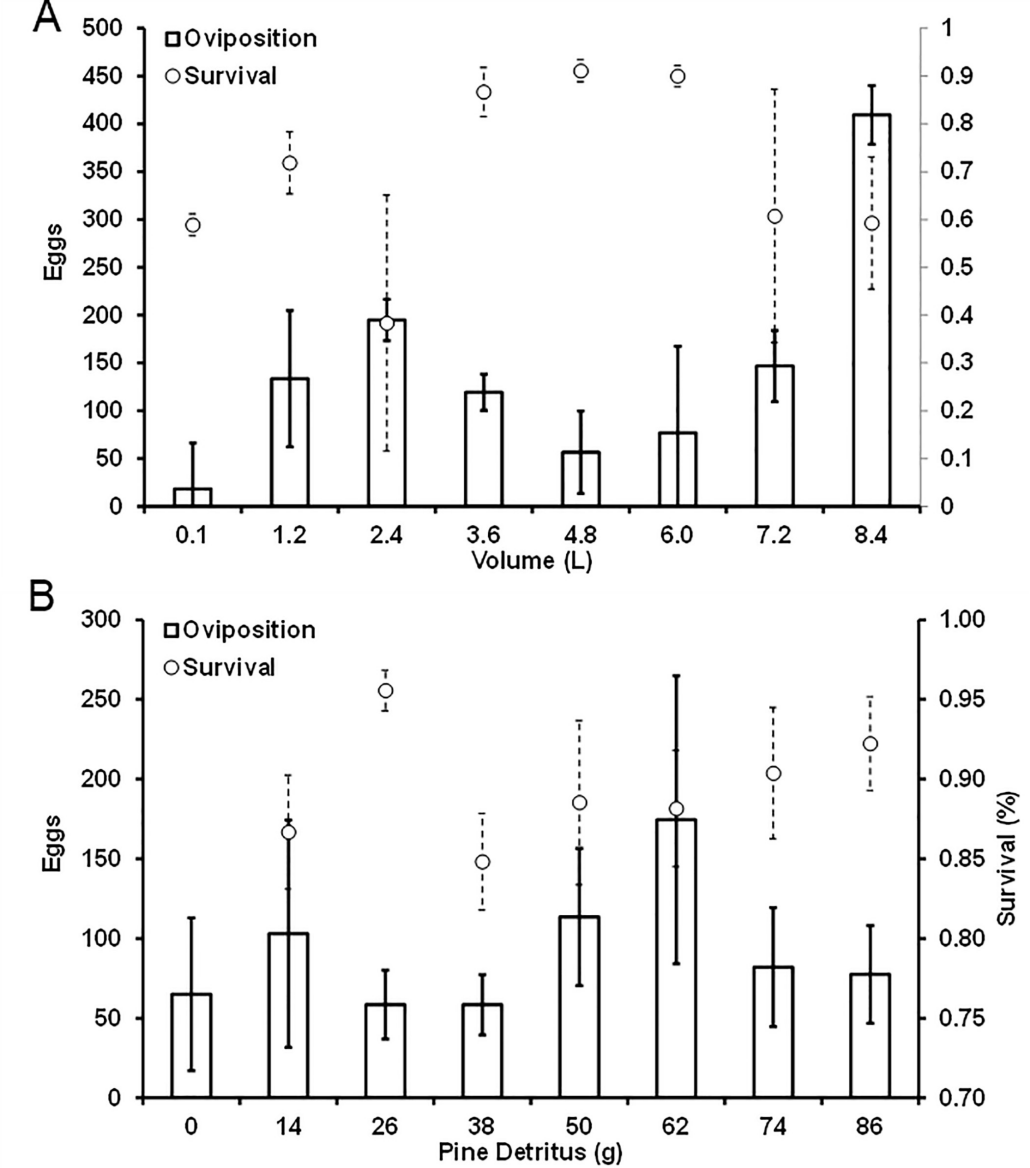
Results (raw means ± SE, dashed for survival, solid for oviposition) from separate experiments examining how environmental factors affect oviposition responses (n = 4 replicates) and larval survival (n = 3 replicates) of *Aedes albopictus*. A. Response to different water volumes in tires, and B. response to different quantities of pine needle detritus.

For *Aedes albopictus*, neither the number of eggs laid (F_7,21_ = 1.78, P = 0.144) nor larval survival (F_7,22_ = 1.95, P = 0.131) varied across different volumes (Fig 1A). On average, 144.5 ± 35.6 eggs were laid per tire and survival across volumes was 71.0 ± 5.2%. In addition, eggs laid (F_7,21_ = 1.22, P = 0.336) and larval survival (F_7, 22_ = 1.02, P = 0.453) were also not significant across levels of pine detritus (Fig 1B). Females combined to lay 91.6 ± 17.1 eggs per tire, with larval survival across pine on average 89.5 ± 1.4%.

**Fig 2:**
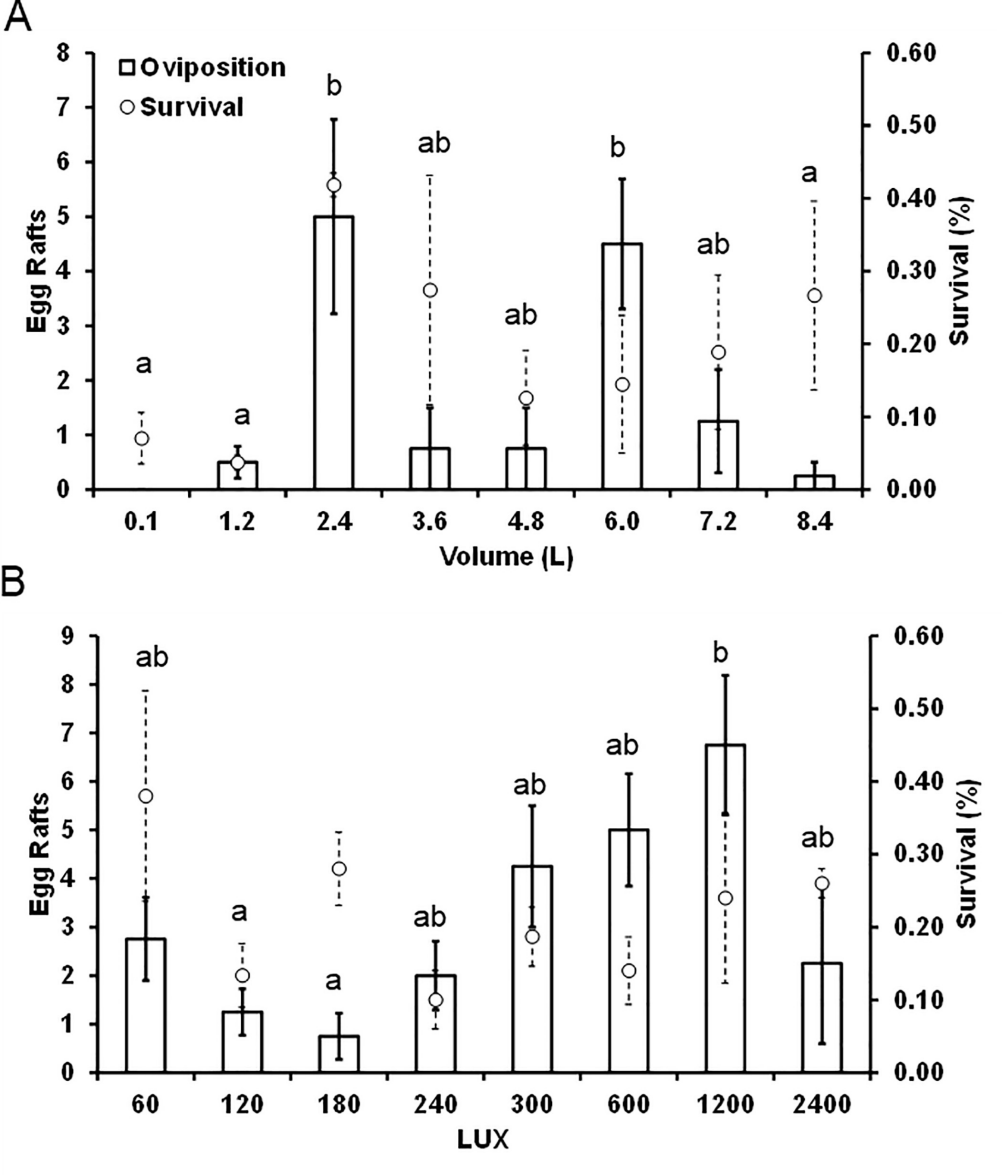
Results (raw means ± SE, dashed for survival, solid for oviposition) from separate experiments examining how environmental factors affect oviposition responses (n = 4 replicates) and larval survival (n = 3 replicates) of *Culex quinquefasciatus*. A. Response to different water volumes in tires, and B. response to different light levels (higher values of LUX are brighter). Means that share a letter are not significantly different at P = 0.05.

For *Culex quinquefasciatus*, there was no effect of volume on larval survival (mean 19.1 ± 3.6%) (F_7, 23_ = 1.84, P = 0.147), however, the number of egg rafts did vary (F_7, 21_ = 3.23, P = 0.017). Specifically, more rafts were laid in the 2.4 and 6.0 L containers compared to the 0.1, 1.2, and 8.4 L, with other volumes intermediate (Fig 2A). For light levels, larval survival also did not vary (mean 21.5 ± 2.9%) (F_7, 23_ = 1.54, P = 0.224), however the number of egg rafts did (F_7,21_ = 3.05, P = 0.022). Specifically, 1200 LUX had more egg rafts compared to either 120 or 180 LUX, with other values being intermediate (Fig 2B).

### Linking oviposition to life history and survival

**Fig 3:**
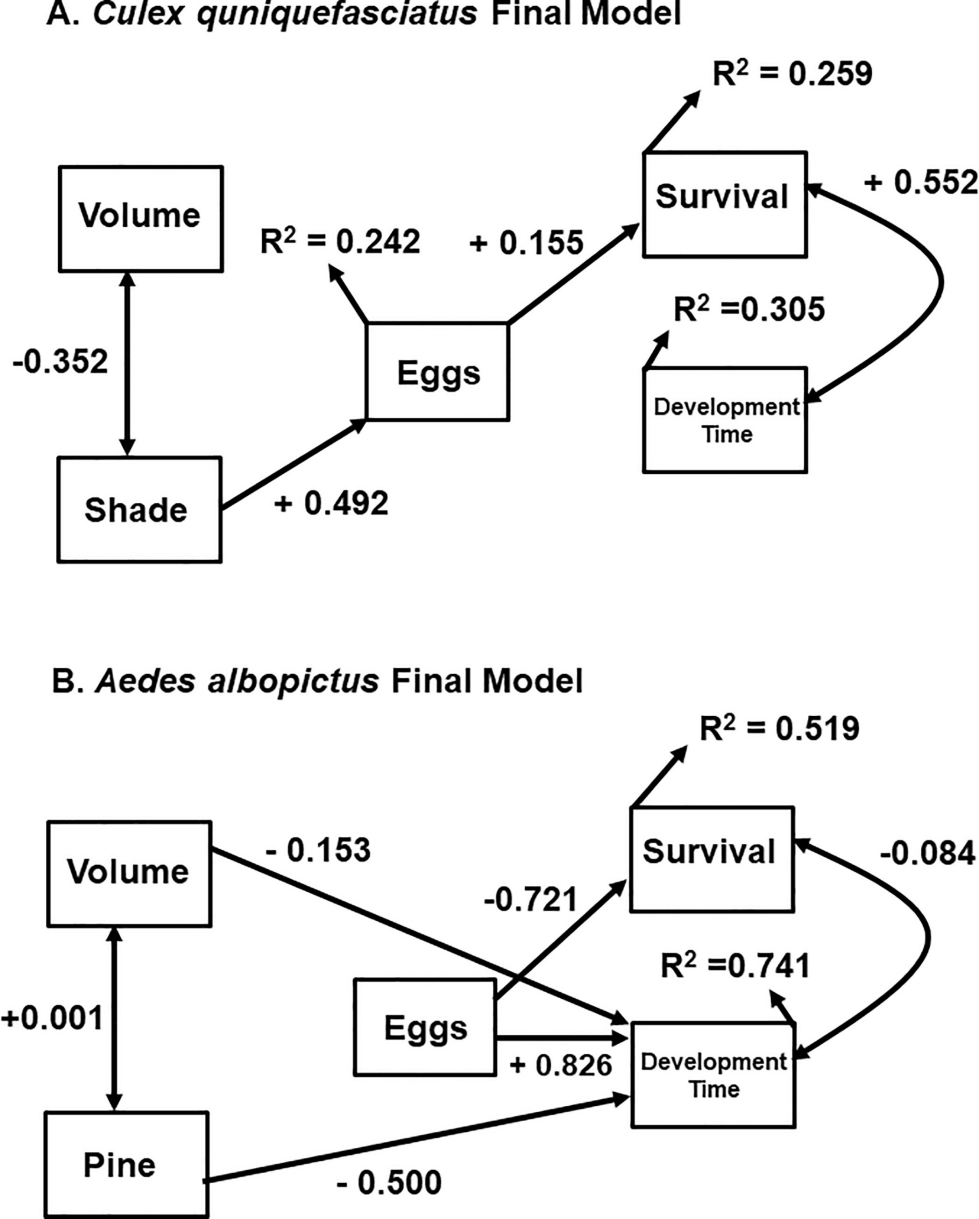
Final path models testing the effect of environmental factors acting as ecological filters on oviposition and larval traits. A. Model for *Culex quinquefasciatus* and B. Model for Aedes albopictus. Values for R^2^ are provided next to each variable along with path coefficients next to each path.

The final model for *Culex quinquefasciatus* resulted in a single variable (shading) affecting the number of eggs produced by each female (Fig 3A). Number of eggs directly affected larval survival in a small but positive way. Survival and development time were positively correlated. Overall, the amount of variation explained in eggs, survival, and development time was generally low. The final model was more consistent with our combined model (connections between environmental factors and eggs laid, as well as eggs and life history traits), however, it lacked connections between independent variables and larval traits (Fig 3A).

**Fig 4:**
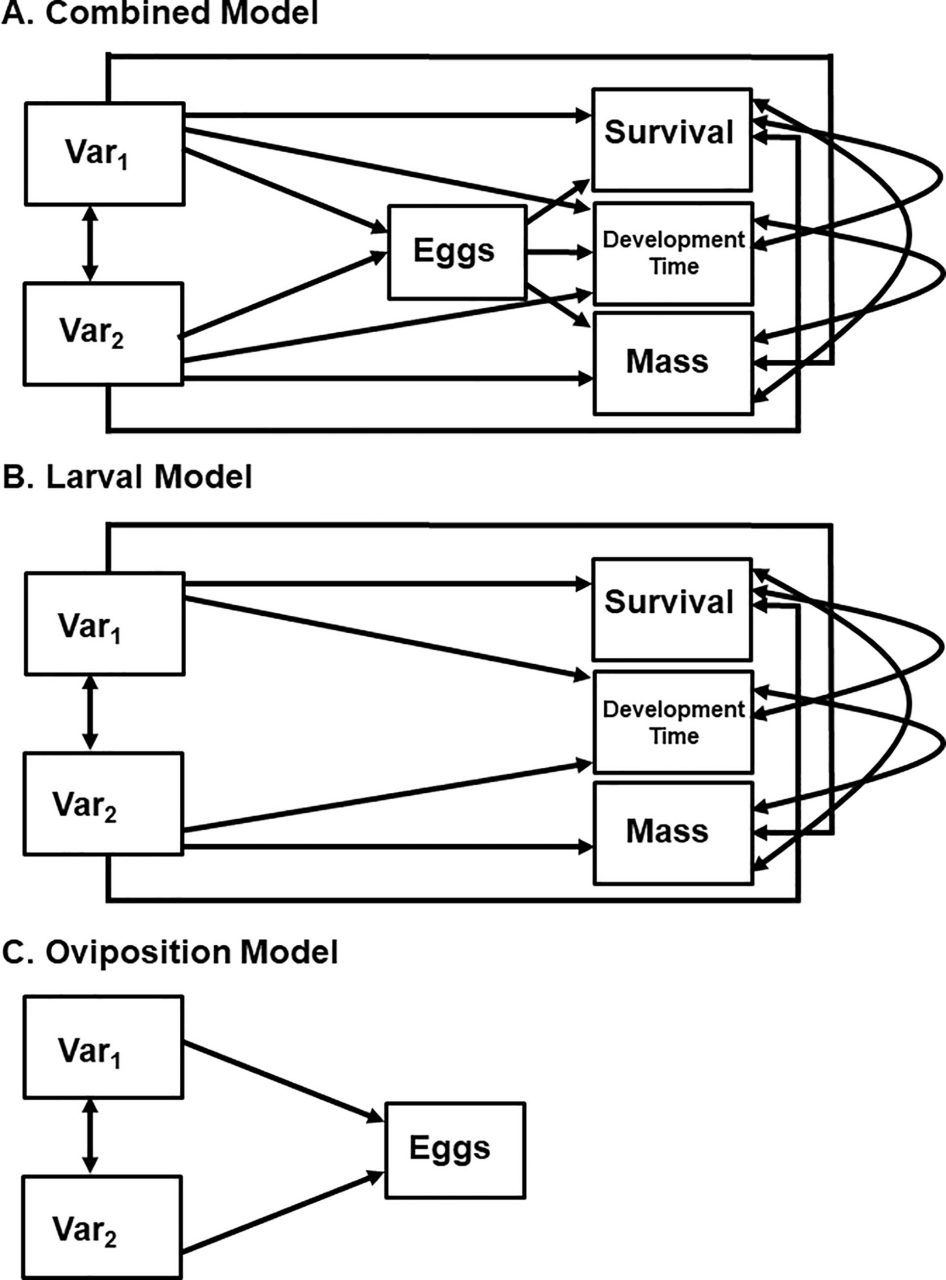
Proposed path diagrams testing the effect of environmental factors (Var_1_, Var_2_) acting as ecological filters on oviposition (eggs) and larval traits (survival, development time, mass). A. Combined/Full model with all links present. B Larval model: no direct links from filters to egg number. C. Oviposition model: no direct links from filters to larval traits.

The final path model for *Aedes albopictus* contained paths between independent variables and most dependent variables (Fig 3B). Specifically, container volume and pine detritus both had a small to moderate negative effect on female development (higher volumes and more pine led to shorter development times). The number of eggs also directly affected larval traits, with more eggs leading to lower survival (negative path coefficient) but longer development times (positive path coefficients); both effects were strong. Finally, there was a weak negative correlation between survival and development time. For both survival and development time, variance explained was moderate to high. The final model was more consistent with our combined model, however, it lacked connections between independent variables and eggs (Fig 4C).

## Discussion

Animals that possess complex life cycles with separate adult and juvenile habitats face a unique challenge in maximizing fitness, as females must possess traits that allow them to accurately assess the quality of potential juvenile habitats and in turn juveniles must have traits that are consistent with those choices. When those traits do not match, then fitness may not be optimal [10, 27]. Herein we tested how oviposition decisions for females and larval survival of two common mosquito species vary across different aquatic environments separately, and then if decisions by females had a long term effect on juveniles. Our results did not support the hypothesis that the success of either species would be a consequence of the combined responses at both the juvenile (larval survival) and adult (eggs laid) stages (e.g., preference-performance hypothesis, [5, 6]). Specifically the environmental factors tested herein did not have the same effect on eggs laid and larval survival for *C*. *quinquefasciatus*, and for *A*. *albopictus* there was no effect of these factors on eggs alone. Although our more parsimonious path models were consistent with the Combined model (which itself would support consistency in environmental factors affecting traits), the model for each species lacked important connections (i.e., environmental factors and eggs in *A*. *albopictus*, environmental factors and larval parameters for *C*. *quinquefasciatus*). This suggests a disconnect in responses of different stages to different degrees; such disconnects between responses of females and their offspring to similar environmental factors would imply that patterns of populations or communities of species with complex life cycles are not so easily predicted.

To date, there has been a greater emphasis on the role of biotic factors [15, 26] than in understanding the role of the abiotic environment in shaping communities of mosquitoes occupying small aquatic containers, like tree holes and discarded vehicle tires (summarized in [51]). Herein we evaluated the independent and combined effects of environmental factors on oviposition and larval traits in two ecologically and medically important mosquito species. Overall, our models provided contrasting evidence for how the environmental factors we manipulated affected each mosquito species. For *C*. *quinquefasciatus*, the number of eggs laid was affected by shading, with tires receiving more light receiving more eggs than shaded ones. Egg number independently went on to affect, in a small but positive way, the number of surviving larvae. This pattern fits with part of the preference-performance hypothesis via the direct effects on female oviposition traits, but is not consistent for an effect on larval survival, as this latter variable was only indirectly affected by egg number. Patterns for larvae in containers are affected by canopy cover for several *Culex*, including *C*. *quinquefasciatus* (e.g., [52, 53]) that often prefer sunlit over shaded habitats. This result is consistent with our independent experiments examining shading and volume only, where light levels affected egg rafts but had no effect on larval survival.

For *Aedes albopictus*, our path analyses revealed that egg numbers were independent of volume and pine detritus, implying that females were not being influenced by these environmental factors when selecting among containers. This is also consistent with our individual trials where neither of these factors affected egg laying. Two explanations here seem likely, that either females were indiscriminate with their egg laying, or that the levels of pine and volume were all equally suitable to gravid females. The latter explanation does not fit with the fact that we used data from a previous study [38] that showed strong variation in larval abundance across the same range of environmental variables. Thus, we are left with females laying eggs across all habitats equally regardless of quality. This is consistent with a general behavior of some mosquitoes (and some *Aedes* specifically) known as skip oviposition [54], where females spread their eggs from a single gonotrophic cycle across multiple habitats. Laboratory and field studies have shown that *A*. *albopictus* [54] and *A*. *aegypti* [55, 56] do display skip oviposition behavior. Specifically, gravid female *A*. *albopictus* usually select the best quality habitat [57], but often distribute eggs across similar quality habitats, a bet-hedging strategy noted for other animals elsewhere [14]. In addition, Fonseca et al. [58] found that *A*. *albopictus* females became less choosy during the summer, as the risk of an suboptimal decision is less compared to winter conditions.

We did note that one measure of larval life history for *A*. *albopictus*, development time, was affected by both environmental factors directly. The high variance explained in development time by the model was therefore supportive that life history alone was affected by environmental factors. In addition, both survival (negative) and development time (positive) were directly affected by egg number. Thus, although the numbers of eggs laid were not a consequence of particular environmental factors, the initial number of eggs did have a direct effect on the life history of larvae. This seems to suggest that initial cohort density is more important in affecting these variables. There is evidence that intraspecific densities affect *A*. *albopictus* but at a rate lower than interspecific competitive effects; however, this is often context dependent [15, 59]. Studies that have examined intra- vs. interspecific competition in mosquitoes have shown that *A*. *albopictus* is the superior resource competitor [59], however, fewer studies have examined intraspecific effects in isolation. Ellis [43] examined density-dependence effects in relation to oviposition and the preference-performance hypothesis for another container species, *Aedes* (*Ochlerotatus*) *triseriatus*, and found that density and habitat quality (measured in terms of detritus type) interacted to affect oviposition patterns. However the work by Ellis [43] had containers of varying quality nested within two habitats (evergreen and deciduous forests), unlike our work where containers were found within the same matrix, making direct comparisons difficult. The fact that egg numbers in our work did not vary among levels of environmental factors, but that they did have strong effects on life history does point to density dependence as an overlooked factor in affecting this species [58].Egg aggregation or dispersion occurs in many species of animal (e.g., [60]). Dispersion may occur to prevent intraspecific competition that may reduce future offspring success, whereas aggregation may increase fitness via reduced predation (e.g., [61]) or other methods to promote progeny survival (e.g., group feeding). Broadly speaking, our results imply that *A*. *albopictus* exhibits egg dispersion (skip oviposition) whereas *C*. *quinquefasciatus* exhibits aggregation. *Aedes albopictus* displays negative density dependent oviposition [16, 58], wherein once eggs are laid in preferred habitats, subsequent females lay eggs in habitats that are likely less productive for larvae. It is hypothesized that negative density dependence can alleviate intense intraspecific competition among *A*. *albopictus* larvae [15, 62]. We found support for this, as eggs were laid in all habitats regardless of detritus amounts or volume. Also, our path analysis failed to identify environmental factors as important for egg laying, which seems to imply that instead of a lack of choice among available habitats, females of this species were acting in a density dependent fashion, thereby spreading eggs among all habitats. Such behavior, if consistent across a wide set of venues, may help explain the success of this highly invasive species across the globe [58]. However, path analyses did show that larval life history traits were also affected by environmental factors, with direct effects on development times. Therefore, although environmental factors did not have consequence for eggs laying, it did affect factors related to success of larvae, and thus indirectly on fecundity of females. This disconnect between larvae and adult traits is an important but understudied aspect of mosquito populations, but has been found to occur in *C*. *quinquefasciatus* under different conditions [16].

In contrast, to the density dependence observed in *A*. *albopictus*, *C*. *quinquefasciatus* eggs showed a clumped pattern in containers with moderate sunlight and larger volumes. *Culex quinquefasciatus* has been shown to aggregate eggs based on responses to an aggregation pheromone, which may interact in unpredictable ways with other stimulatory oviposition cues to affect patterns of oviposition [63, 64]. In this species, larvae may benefit from egg aggregation, as larvae are filter-feeders and obtain nutrition from floating microorganisms and dissolved nutrients [25, 30]. Thus, we may predict that higher densities of larvae may facilitate feeding efficiency, however higher crowding can lead to development delays [65]. Our path analysis did not identify any links between environmental factors and development time, or eggs and development time. This may be because densities of larvae in tires were not high enough to elicit the effects of crowding reported by others. The densities we observed were driven by female oviposition choice, and even our experiments set to examine effects of environmental factors on survival were based on natural densities of larvae in tires. Thus, it appears more likely that our experiments have identified a disconnect between oviposition and larval survival. One intriguing explanation for this disconnect is that *C*. *quinquefasciatus* uses open water habitats as a primary source of larval development [16], although it is a common member of the container mosquito community [52]. If females have evolved to use cues related to open water habitats (e.g., surface area, water reflectance), then they may be confusing larger containers with open water habitats. This would be in spite of a range of containers being able to support their larvae, a fact we found when exploring the response to environmental factors separately.

We note some limitations of the work presented here. First, our work was specific to mosquito systems, and it has been noted that PPH has had variable support for other insects, including those that feed directly on plants [e.g., 66, 67]. Second, in some instances we gave larvae different diets under different treatments (i.e., *Aedes albopictus* in pine were given plant detritus whereas those across volumes were given animal detritus). Given that animal and plant detritus are known to have significant effects on larval mosquito development (e.g., [68]), this may have affected some of our results. However, we note that survival of *Ae*. *albopictus* was generally higher in the poorer plant resources (mean survival ± SE, pine 89% ± 0.1%, animal detritus in volume 71% ± 0.5%), and our path analysis included both factors in affecting life history traits. This suggests that although detritus likely affected some traits, the broad patterns we identified were robust to the factors we manipulated. Third, our experiments were restricted to our temperate/semi-tropical location in southern Mississippi. However, both of our focal species have a world-wide distribution, and as they would experience different climates and may have a greater variety of habitat types (e.g., [69, 70]), our results may or may not be applicable everywhere. Thus, it is important for the an assessment of local conditions across a wider range of conditions to get a fuller understanding of the PPT for these species. Finally, the sample size of our combined experiment may have affected our ability to identify important paths in our models. In some cases, dozens or hundreds of replicates are necessary to achieve adaquate power in structural equation models, however the number of sufficient replicates is affected by the number of proposed paths, the number of latent variables, and the number of dependant variables [71]. Regardless, we do note that the amount of variation explained by our model was often high, especially for *Culex quniquefasciatus*, and our findings for both species were broadly consistent with other studies for these species (e.g., [21, 28, 38, 44, 58]).

Egg laying decisions have important consequences for population dynamics and spatial distributions of species, however examinations of oviposition behavior and larval distribution may be misleading. For instance, efforts to control the yellow fever mosquito (*Aedes aegypti*), a species that uses container habitats, have failed when targeting only the most productive larval habitats [72], as adult females will lay eggs in non-preferred container types that are often equally viable or more suitable for offspring performance when the preferred larval habitats are absent [72, 73]. Thus, although source reduction in juvenile habitats may be a short term method to control mosquitoes of medical importance, it may unintentionally cause a shift in oviposition behavior without drastically affecting population dynamics. Moreover, disconnects in female egg-laying decisions and larval performance may indicate that population dynamics for species with complex life cycles may be harder to predict.

## Supporting information

S1 Table Summary of the number of instances when environmental factors were identified as important in significant stepwise multiple regression analysis for *Aedes albopictus* and *Culex quinquefasciatus* (data for factors collected in Yee et al. 2015). The number of models where a factor appeared is based on analyses at three scales: 6 counties across 3 time periods, 3 times periods regardless of sites, and the overall data set. Thus, the maximum number of instances that each variable could be present in significant regression models for these scales was 18, 3, and 1. The factors we selected were based on those that were included in 1) the overall model, 2) the most time periods, and 3) the most sites. Factors in bold are those with the highest occurrences in the models and thus used for field and laboratory experiments here.
(DOCX)
