# Correction: Quantifying species traits related to oviposition behavior and offspring survival in two important disease vectors

**DOI:** 10.1371/journal.pone.0250288

**Published:** 2021-04-12

**Authors:** Donald A. Yee, William C. Glasgow, Nnaemeka F. Ezeakacha

The order of Figs 3 and 4 is switched. As a result of the correction, the in-text citations of Figs 3 and 4 are also switched. How the in-text citations of Figs 3 and 4 should be read can be viewed below.

**Fig 3:**
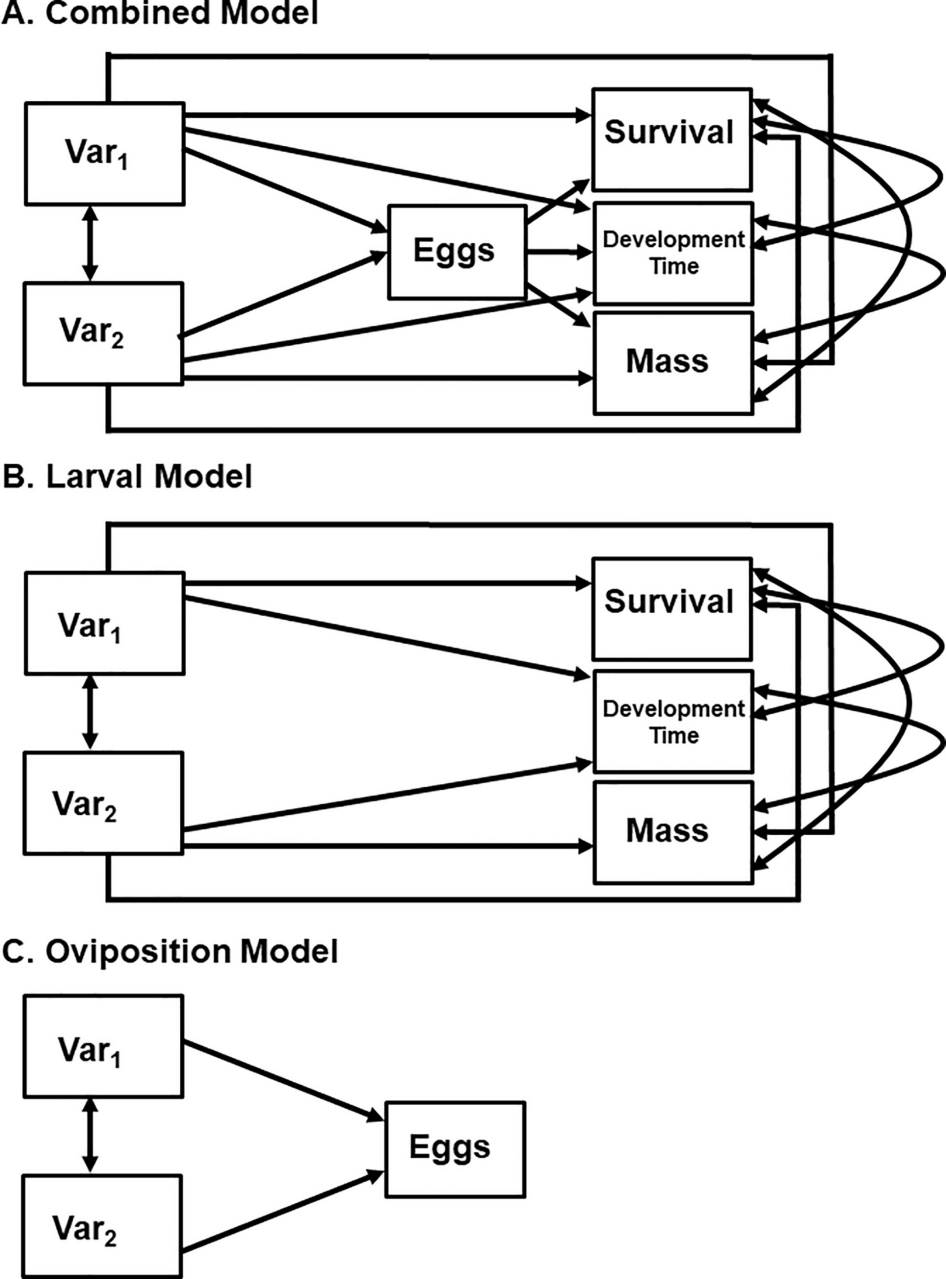
Proposed path diagrams testing the effect of environmental factors (Var_1_, Var_2_) acting as ecological filters on oviposition (eggs) and larval traits (survival, development time, mass). A. Combined/Full model with all links present. B Larval model: no direct links from filters to egg number. C. Oviposition model: no direct links from filters to larval traits.

**Fig 4:**
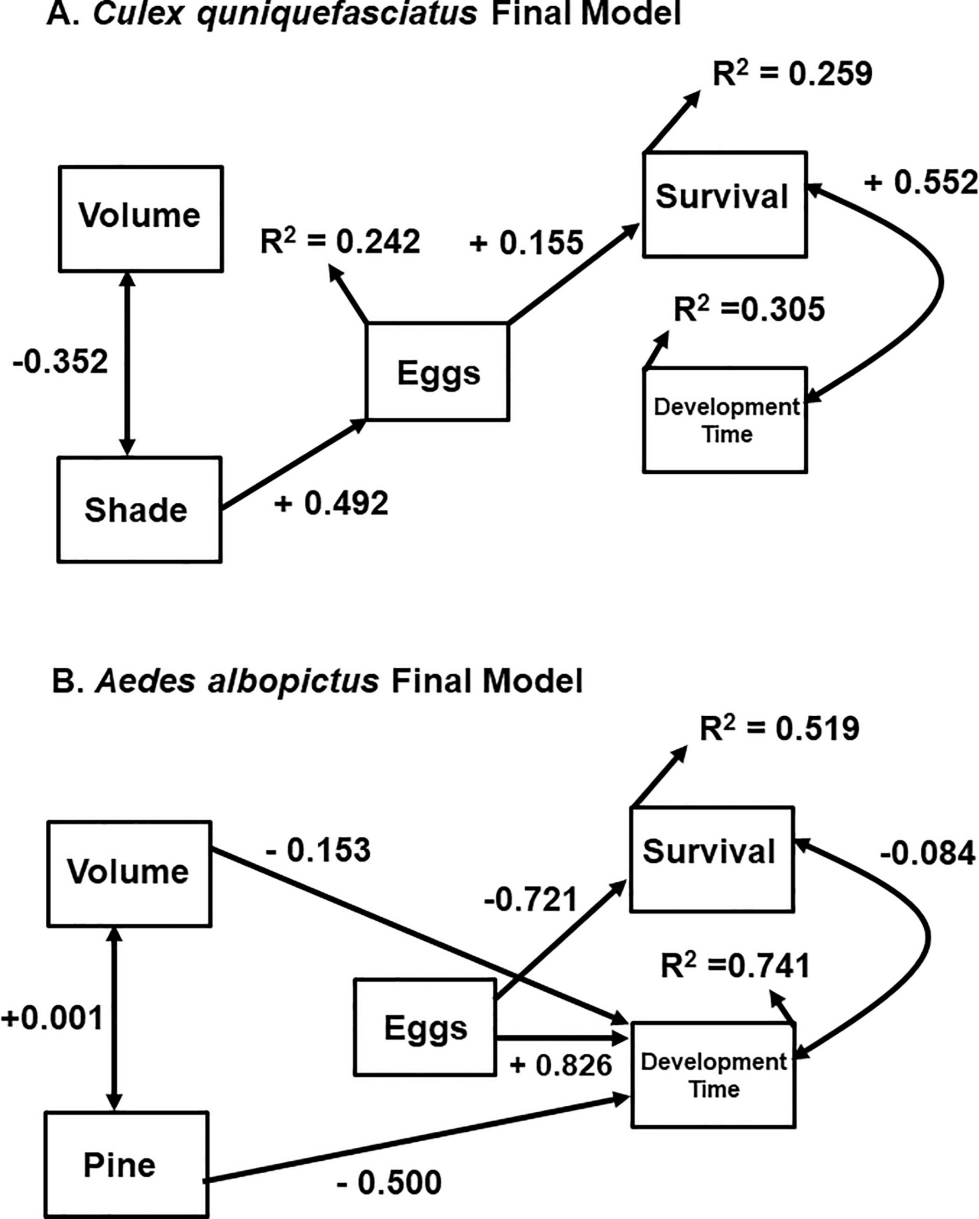
Final path models testing the effect of environmental factors acting as ecological filters on oviposition and larval traits. A. Model for *Culex quinquefasciatus* and B. Model for Aedes albopictus. Values for R^2^ are provided next to each variable along with path coefficients next to each path.

In the Linking oviposition to life history and survival subsection of the Materials and methods, the sixth sentence of the second paragraph should read: We hypothesized three sets of paths based on the relationship between oviposition and larval survival in explaining life history parameters and survival: a Combined Model (Fig 3A), a Larval Model (Fig 3B), and an Oviposition Model (Fig 3C).

In the Linking oviposition to life history and survival subsection of the Results, the first sentence of the first paragraph should read: The final model for Culex quinquefasciatus resulted in a single variable (shading) affecting the number of eggs produced by each female (Fig 4A). The last sentence of the first paragraph should read: The final model was more consistent with our combined model (connections between environmental factors and eggs laid, as well as eggs and life history traits), however, it lacked connections between independent variables and larval traits (Fig 4A).

The first sentence of the second paragraph of the same subsection should read: The final path model for Aedes albopictus contained paths between independent variables and most dependent variables (Fig 4B). The last sentence of the second paragraph should read: The final model was more consistent with our combined model, however, it lacked connections between independent variables and eggs (Fig 3C).
