# Supplementary material for: Quantifying species traits related to oviposition behavior and offspring survival in two important disease vectors
Source: PLoS One. 2020 Sep 25;15(9):e0239636. doi: 10.1371/journal.pone.0239636 (PMC7518596; doi:10.1371/journal.pone.0239636)
Supplement: S1 Table — The number of models where a factor appeared is based on analyses at three scales: 6 counties across 3 time periods, 3 times periods regardless of sites, and the overall data set. Thus, the maximum number of instances that each variable could be present in significant regression models for these scales was 18, 3, and 1. The factors we selected were based on those that were included in 1) the overall model, 2) the most time periods, and 3) the most sites. Factors in bold are those with the highest occurrences in the models and thus used for field and laboratory experiments here. (DOCX) [file pone.0239636.s001.docx]

**Supplemental Table 1**. Summary of the number of instances when environmental factors were identified as important in significant multiple regression analysis for *Aedes albopictus* and *Culex quinquefasciatus* (data for factors collected in [38]). The number of models where a factor appeared is based on analyses of 6 counties across 3 time periods, 3 times periods regardless of sites, and the overall data set. Thus, the maximum number of instances that each variable could be present in significant regression models was 18, 3, and 1. Factors in bold are those with the highest occurrences in the models and thus used for field and laboratory experiments.

| Factor | *Aedes albopictus* | *Culex quinquefasciatus* |
| --- | --- | --- |
| tire diameter | 1, 1, 1 | 0, 1, 0 |
| canopy cover | 1, 1, 0 | **4, 2, 1** |
| water depth | 2, 1, 0 | 1, 2, 1 |
| water volume | **4, 1, 0** | **4, 3, 1** |
| water temperature | 3, 0, 0 | 1, 0, 0 |
| pH | 2, 0, 0 | 2, 1, 0 |
| protozoan richness | 3, 1, 1 | 0, 0, 0 |
| protozoan abundance | 1, 0, 1 | 1, 0, 0 |
| animal detritus | 1, 0, 0 | 0, 0, 0 |
| reproductive detritus | 3, 0, 0 | 1, 1, 0 |
| leaf detritus | 0, 1, 0 | 0, 0, 0 |
| pine detritus | **2, 3, 1** | 1, 0, 0 |
| wood detritus | 3, 1, 1 | 0, 0, 0 |
| fine detritus | 2, 1, 0 | 0, 0, 0 |
